# Quantified Activity Measurement for Medical Use in Movement Disorders through IR-UWB Radar Sensor †

**DOI:** 10.3390/s19030688

**Published:** 2019-02-08

**Authors:** Daehyeon Yim, Won Hyuk Lee, Johanna Inhyang Kim, Kangryul Kim, Dong Hyun Ahn, Young-Hyo Lim, Seok Hyun Cho, Hyun-Kyung Park, Sung Ho Cho

**Affiliations:** 1 Department of Electronics and Computer Engineering, Hanyang University, 222 Wangsimini-ro, Seongdong-gu, Seoul 04763, Korea; ldh166@hanyang.ac.kr (D.Y.); lwh9886@gmail.com (W.H.L.); 2 Department of Psychiatry, Hanyang University Medical Center, 222-1 Wangsimni-ro, Seongdong-gu, Seoul 04763, Korea; iambabyvox@hanmail.net (J.I.K.); kangryul@naver.com (K.K.); 3 Department of Psychiatry, Hanyang University College of Medicine, 222 Wangsimini-ro, Seongdong-gu, Seoul 04763, Korea; ahndh@hanyang.ac.kr; 4 Division of Cardiology, Department of Internal medicine, Hanyang University College of Medicine, 222 Wangsimni-ro, Seongdong-gu, Seoul 04763, Korea; mdoim@hanyang.ac.kr; 5 Department of Otorhinolaryngology-Head and Neck Surgery, Hanyang University College of Medicine, 222 Wangsimni-ro, Seongdong-gu, Seoul 04763, Korea; shcho@hanyang.ac.kr; 6 Department of Pediatrics, Hanyang University College of Medicine, 222 Wangsimni-ro, Seongdong-gu, Seoul 04763, Korea; 7 Hanyang Inclusive Clinic for Developmental Disorders, Hanyang University Medical Center, 222-1 Wangsimni-ro, Seongdong-gu, Seoul 04763, Korea

**Keywords:** IR-UWB radar sensor, movement disorder, hyperactivity, actigraphy

## Abstract

Movement disorders, such as Parkinson’s disease, dystonia, tic disorder, and attention-deficit/hyperactivity disorder (ADHD) are clinical syndromes with either an excess of movement or a paucity of voluntary and involuntary movements. As the assessment of most movement disorders depends on subjective rating scales and clinical observations, the objective quantification of activity remains a challenging area. The purpose of our study was to verify whether an impulse radio ultra-wideband (IR-UWB) radar sensor technique is useful for an objective measurement of activity. Thus, we proposed an activity measurement algorithm and quantitative activity indicators for clinical assistance, based on IR-UWB radar sensors. The received signals of the sensor are sufficiently sensitive to measure heart rate, and multiple sensors can be used together to track the positions of people. To measure activity using these two features, we divided movement into two categories. For verification, we divided these into several scenarios, depending on the amount of activity, and compared with an actigraphy sensor to confirm the clinical feasibility of the proposed indicators. The experimental environment is similar to the environment of the comprehensive attention test (CAT), but with the inclusion of the IR-UWB radar. The experiment was carried out, according to a predefined scenario. Experiments demonstrate that the proposed indicators can measure movement quantitatively, and can be used as a quantified index to clinically record and compare patient activity. Therefore, this study suggests the possibility of clinical application of radar sensors for standardized diagnosis.

## 1. Introduction

Movement disorders, such as Parkinson’s disease, dystonia, tic/Tourette’s disorder, and attention-deficit/hyperactivity disorder (ADHD), are clinical syndromes with either an excess of movement or a paucity of voluntary and involuntary movements. The assessment of many movement disorders has heavily relied on clinical observation and rating scales, which are inherently subjective, and results vary according to the informant [1]. There is an increasing need for tools that objectively evaluate the level of activity. We focused on ADHD, among various movement disorders, to explore the possibility of a new evaluation method. ADHD is a common neurodevelopmental disorder characterized by inattention, impulsivity, and hyperactivity [2]. In contrast to the research on objective measurements of inattention, such as the continuous performance test (CPT), the assessment of hyperactivity in clinical settings is based on subjective reports from caregivers and from the observations of clinicians [3]. As hyperactivity is influenced by environmental factors and cognitive demands, discrepancy regarding the description of hyperactivity often occurs, thereby making the diagnosis of ADHD challenging [4].

Studies have been conducted, using infrared cameras (QbTest; Qb Tech, Stockholm, Sweden), 3D cameras (Microsoft Kinect, Redmond, US), or actigraphy (ActiGraph, Florida, US), to measure the objective level of activity in young people having ADHD [5]. However, these sensors have not been applied widely in clinical settings due to several limitations. A recent study reported that the QbTest is insufficient as a diagnostic test for ADHD, as it is unable to differentiate ADHD from other neurodevelopmental disorders [6]. Examination methods using infrared cameras, such as QbTest, are not perfectly non-contact, and measure the patient’s concentration, but do not measure activity [7]. It can be difficult to judge exact whole-body motions, as these methods reflect only the movement of a specific part of the body. In the case of a depth camera or a 3D camera, the angle of view is limited to about 60 degrees, the performance varies depending on the indoor lighting environment, and the maximum measurable distance is as short as several meters [8]. Actigraphy has been the most commonly used device in measuring hyperactivity in ADHD [9]. Its primary use is measuring sleep and wakefulness, but it can also measure the movement of the subject in the *x*, *y*, and *z* axes, through an acceleration sensor [10]. It is not only possible to measure the amount of activity by obtaining the number of steps and the vector magnitude with this acceleration data, but the position can be estimated (even though the error is cumulative). However, as the device is worn on a certain part of the body, such as ankles and wrists, activity measurement does not reflect the movements of the whole body. The device is attached to the skin, and it may cause inconvenience for the user. Currently, actigraphy is considered to be useful in monitoring motor activity during treatment, but there is little evidence supporting the use of actigraphy in the diagnosis or as a screening tool for ADHD [11].

Impulse radio ultra-wideband (IR-UWB) radar sensors are capable of detecting objects without interference from other sensors through the use of ultra-wideband frequencies. Despite sending and receiving signals with very low power to comply with Federal Communications Commission (FCC) standards, they have enough range and resolution to observe the indoor environment. The primary advantages for clinical application of an IR-UWB radar sensor are its very low power and high spatial resolution. IR-UWB radar signals typically have a high resolution, so it can be used to detect the fine motion of objects [12]. Moreover, it is harmless to the human body and enables the diagnosis of the subject by a non-contact method, causing no inconvenience for the patient. The radar sensor is in a sustainable form and has no contact or requirement for the patient. As it has excellent penetrability, it can be installed on the wall invisibly, and so it is able to observe the target without attracting any attention from the target. Due to these characteristics, the measurement and quantification of activity in clinical movement disorders using an IR-UWB radar sensor is very promising. The IR-UWB radar sensor is capable of detecting not only large movements of the human body but also small movements, such as breathing. Recently, communications, localization, positioning, and tracking using the IR-UWB radar sensor have been studied. Most applications can be performed simultaneously using the same hardware [13,14,15].

The purpose of this study was to calculate the objective quantity of movement by using four radar sensors to find the position of the subject, and to calculate the amount of body movement in a testing room, during an attention task called the comprehensive attention test (CAT), which is a computerized CPT widely used for ADHD patients [16]. Through this study, we will quantify the movement of subjects and present new indicators that can potentially be applied in the measurement of activity in movement disorders, such as ADHD. All of the different radar functions have one thing in common: The information is based on human movement. Therefore, movement information was obtained from radar signals and two types of movements were defined which could be measured by radar, based on changes in position. In regard to spatial movement (which refers to the movement of the subject accompanied with position change), the degree of movement can be calculated by replacing the position change amount with a vector by tracking. In regards to sedentary movement (which refers to the movement of the subject accompanied by little or no position change), the degree of movement was calculated by continuously measuring the amount of change in the magnitude of the reflected signal from the target.

The following sections introduce the signal model of the IR-UWB radar and the basic concept of the algorithm for the activity measurement. Then, a detailed description of the algorithm, based on the tracking and signal magnitude, is presented. Finally, after introducing the experimental environment and methods, experimental results are presented and analyzed.

## 2. Problem Statement

### 2.1. Signal Model and Basic Signal Processing

The impulse signal s[k], emitted by the radar to observe the target area, is delayed and scaled while being reflected from the surrounding environment. The received signal of the radar is generated by the reflected s[k] through $N_{path}$ paths from the surrounding environment. The signal received by the i-th radar can be represented by the sampled signal $x_{i}\left[k\right]$, including the environment noise N[k], as follows

(1) $$x_{i}\left[k\right]=\sum_{m=1}^{N_{path}}a_{m,i}s\left[k-\tau_{m,i}\right]+N\left[k\right].$$

The sampled time index *k* can be called a distance index, and is represented by a natural number from 0 to $L_{signal}$, which is the distance index of the maximum observable distance. When s[k] is reflected on the *m*-th path of the *i*-th radar, $a_{m,i}$ and $\tau_{m,i}$ are the scale values and delays, respectively [15].

**Figure 1:**
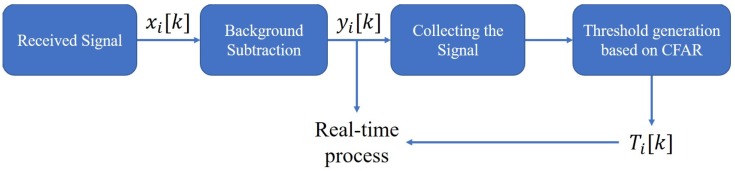
Basic signal processing.

In an indoor environment, there are a lot of objects and walls and so there are various signals received, in addition to people. Reflected signals from the background are called clutter signals, and usually have a large and constant magnitude. Removing the clutter signals is necessary to observe only the reflected signal from the target, and requires a detection algorithm to detect people while excluding noise. Hence, we can only obtain signals for the target in the indoor environment. The basic signal processing procedure for detecting people is shown in Figure 1.

Background removal algorithms are used frequently in indoor environments to observe only the desired targets. A signal $y_{i}\left[k\right]$ with background removed from the received signal $x_{i}\left[k\right]$ can be obtained. The purpose of the initialization phase is to create a threshold. This threshold should reflect the characteristics of the experimental environment, so the initialization should proceed without any humans in the observation area of the IR-UWB radar. The environment-adapted threshold $T_{i}\left[k\right]$ is, then, used for detection in the real-time process.

Background subtraction is a technique for separating the foreground from the background, where walls or static objects correspond with the background, while the observed target corresponds with the foreground [17]. With this algorithm, background signals can be removed, and only the signal components of a moving target can be detected. The background clutter signal $C_{i,n}\left[k\right]$ is continually updated from the previous clutter signal $C_{i,n-1}\left[k\right]$ and $x_{i,n}\left[k\right]$, where *n* is the sequence number of the received signal in each radar, and $C_{i,n}\left[k\right]$ is subtracted from the radar signal $x_{i,n}\left[k\right]$ to obtain the background subtraction signal $y_{i,n}\left[k\right]$, which is expressed as

(2) $$\begin{array}{cc} y_{i,n}\left[k\right] & =x_{i,n}\left[k\right]-C_{i,n}\left[k\right],\\ C_{i,n}\left[k\right] & =\alpha C_{i,n-1}\left[k\right]+\left(1-\alpha \right)x_{i,n}\left[k\right]. \end{array}$$

To more accurately detect the signal of the target, the distance between the subject and the radar can be calculated from the background subtraction signal using the Constant False Alarm Rate (CFAR) algorithm [18]. Generally, it is common to detect using the cell-averaging (CA-CFAR) method with a certain window size in one-frame data received from the radar. However, the $y_{i}\left[k\right]$ collected by observing the environment without a target for a certain duration is represented as $Y_{i}\left[k\right]={\left[y_{i,0}\left[k\right],y_{i,1}\left[k\right],y_{i,2}\left[k\right],\cdot \cdot \cdot ,y_{i,N_{c}}\left[k\right]\right]}^{T}$, and is used for threshold value-generation, based on the CFAR method, where $N_{c}$ is the number of collected $y_{i}\left[k\right]$. This allows the probability of false alarms to be set to a certain value by comparing the received signal from the target with the threshold level $T_{i}\left[k\right]$, expressed as

(3) $$T_{i}\left[k\right]=\beta \;\sigma_{i}\left[k\right]+\mu_{i}\left[k\right],$$

where the subscript *i* points to the *i*-th radar, β is a parameter to adjust the false alarm rate, and $\mu_{i}\left[k\right]$ and $\sigma_{i}\left[k\right]$ are the mean and standard deviation of $Y_{i}\left[k\right]$, respectively. When the background signal is removed, only the target signal and the noise remain, and the $y_{i}\left[k\right]$, applied with the background subtraction algorithm, can be expressed as

(4) $$y_{i}\left[k\right]={\hat{r}}_{i}\left[k\right]+N_{i}\left[k\right],$$

where ${\hat{r}}_{i}\left[k\right]$ is the target signal estimated by the clutter removal in *i*-th radar and $N_{i}\left[k\right]$ is the noise [15]. Therefore, if there is no target, such as when collecting a signal for a threshold, $y_{i}\left[k\right]$ only has noise. To detect the target separately from the noise, we can obtain the mean and variance of $Y_{i}\left[k\right]$, and create a threshold as shown in Equation (3).

### 2.2. Basic Concept of Activity Measurement

Generally, because the reflection coefficient of the electromagnetic wave to the target does not change, there is no change in $x_{i}\left[k\right]$ if there is no movement. Conversely, when there is movement, the value of $x_{i}\left[k\right]$ changes because some paths differ from those of the previous environment. Additionally, the distance measurement to the target is calculated as *k*, where $x_{i}\left[k\right]$ is largely changed. The distance resolution of the UWB radar has units of a few millimeters, which can detect very small changes within the range of the radar. Therefore, it is impossible for a radar to miss even the very small movements of a human, and the amount of activity of the target can be measured by the change of the magnitude of the radar signal and the moving distance information. However, because the radar can measure only one-dimensional distance data, it is limited to observing the target with one radar. Thus, multiple radars were used to measure the position of the target while simultaneously measuring the change in the magnitude of the signal, which was represented as activity.

In this paper, human movement is divided into two types: A type with a change in position, and a type with no change in position (such as sitting). The former is defined as spatial movement, and the latter is defined as sedentary movement. In the past, these two movements have been measured in different ways. One way is to measure the target’s motion intensity (e.g., actigraphy sensor [19]), and the other is to track the target [5]. These two modes of motion are independent of each other, and have different characteristics. Spatial movements are observable movements from a macroscopic point of view, and sedentary movements are observable movements from a microscopic point of view. If the position of the target does not change, the measured distance of the radar does not change significantly, so the positioning information will not reflect sedentary movements well [20]. Conversely, if there is a change the position, the positioning information may reflect this movement, but it is difficult for the received signal magnitude to reflect the movement state, such as the position change or movement speed. Therefore, because it is difficult to confirm the amount of activity in all cases with one measurement method, an algorithm is proposed in this paper that can numerically compare the amount of activity through two indicators.

### 2.3. Experiment Scenario

We designed several scenarios to check our proposed indicators. The criteria for dividing the scenario first broadly, based upon whether there is any spatial movement, divides scenarios into two groups. These groups are then divided into several scenarios, depending on the degree of movement. This study is not intended to recognize specific actions, because it is aimed at measuring movement by projecting human motion using one-dimensional data. Therefore, each scenario was designed to include random behavior with minimal limitations. The list of scenarios is as follows:

1. When a person sits and concentrates on one thing;
2. When a person has a relatively small motion in a sitting position;
3. When a person has a relatively large motion in a sitting position;
4. When a person walks slowly in the room in a narrow radius;
5. When a person walks slowly in the room in a large radius;
6. When a person walks quickly in the room in a narrow radius;
7. When a person walks quickly in the room in a large radius.

The scenarios were designed to account for situations including movement during the test, and were only for reproducing other test environments. The proposed indicators have no particular dependency on CAT. Scenario 1 is a situation in which the target is focused on the test and does not move. In this scenario, the subject should minimize any actions other than the restricted, small movements required for the test. Scenarios 2 and 3 assumed that the target was seated for CAT, but cared about other things. Scenario 2 is a situation in which the limbs and the head move while the torso is fixed (such as looking around or touching something else). Scenario 3 is a situation in which the entire body moves in a sitting position, such as sitting with the chair tilted back. Scenarios 4–7 are four scenarios created using two opposing features. Scenarios 4 and 6 include walking near the center of the room, while Scenarios 5 and 7 include roaming the entire room. While Scenarios 4 and 5 are relatively slow walking scenarios, Scenarios 6 and 7 are relatively fast walking scenarios.

The greater the torso movement, the greater the sedentary movement index. This is because the torso takes up most of the human body. Thus, for our scenarios, the size of the sedentary movement index can be expected to decrease in order of Scenarios 3, 2, and 1. In Scenarios 4–7, it was expected that walking around a wide area or moving quickly would be observed as a larger movement than walking around a narrow area or moving slowly.

## 3. Algorithm for Measuring Activity

### 3.1. Measuring the Sedentary Movement

If there is a person with any movement, the corresponding $y_{i}\left[k\right]$ deviates greatly from the probability characteristics of the vacant state, so a person can be easily detected by comparing $y_{i}\left[k\right]$ with the threshold $T_{i}\left[k\right]$. If there is no person at the position of the *k*-th sample for *i*-th radar, ${\hat{r}}_{i}\left[k\right]$ is estimated to be zero in Equation (4), and $y_{i}\left[k\right]$ is not helpful in measuring activity. Previously, movement was simply represented as the sum of the differences in signal amplitude [21,22]. In this case, however, even when the difference between two consecutive frames is obtained, noise cannot be reduced. In cases where the motion is small, the amplitude of the target signal can be reduced. Therefore, to make only the signals from the target into activity indicators, we only used the samples for *k* where $y_{i}\left[k\right]$ exceeds the threshold $T_{i}\left[k\right]$, which can be expressed as

(5) $$E_{i}\left[n\right]=\sum_{k=0}^{L_{signal}}g_{i,n}\left[k\right],\;g_{i,n}\left[k\right]=\left\{\begin{array}{cc} {\left|y_{i,n}\left[k\right]-y_{i,n-1}\left[k\right]\right|}^{2}\; & if\;y_{i,n}\left[k\right]>T_{i}\left[k\right]\\ 0\; & if\;y_{i,n}\left[k\right]\leq T_{i}\left[k\right] \end{array}\right..$$

Of course, the received signal of a radar differs greatly, according to the distance to the target, and so, for a single radar, the degree of movement will vary greatly depending on the position. However, to compensate for this difference, it is necessary to consider not only the attenuation compensation along the distance, but also the compensation according to the antenna pattern in three dimensions. Further studies are needed to consider the relationship between position dependent signal attenuation and the clutter cancelling signal. We used the median of the data obtained by installing four radars at each corner of the four directions to apply the minimum compensation. If the target is too close to (or far from) any radar, it will be measured as too large (or too small), so the maximum and minimum values of $E_{i}$ are excluded. Therefore, the proposed indicator to observe the sedentary movement can be expressed as

(6) $$M_{sedentary}\left[n\right]=\mathrm{Median}\left(E_{0}\left[n\right],E_{1}\left[n\right],E_{2}\left[n\right],\cdot \cdot \cdot ,E_{N_{r}}\left[n\right]\right),$$

where $N_{r}$ is the number of radars for measurement and Median(·) is the function that returns the median value of the input value.

### 3.2. Measuring the Spatial Movement

There are not many people who move at a constant speed or only perform one action. Human behavior varies, and human movement is closely related to many forces; examples include friction forces and reaction forces from the earth. Due to these forces, the human movement state is constantly changing. However, for people who are not moving, the only movement is due to breathing. This means that there is almost no change in force. Because the force that moves a target is represented by the acceleration of the target, a person with active motion will have a greater acceleration than a person with slight motion. For this reason, it is possible to measure the amount of activity of an object through actigraphy [9]. As we cannot mathematically model the random movements of a person, we can use numerical differentiation to obtain the acceleration value from the data measured. Although the accuracy of the calculated acceleration may be low, we do not need the exact value [23].

The process of obtaining the acceleration begins by obtaining the distance value from each radar signal $y_{i}\left[k\right]$, obtained in Section 2.1. Methods for distance measurement in radar are already well known [24]. When the target exists in the observation region, multipath causes the signal magnitude to change at the distance index behind the target signal [15]. This magnitude change can be sufficiently above the threshold. Hence, in the signal $y_{i}\left[k\right]$, the shortest distance from the radar to the target can be obtained by using the minimum value of *k* satisfying $y_{i}\left[k\right]>T_{i}\left[k\right]$. Using the sampling frequency of the radar to convert from *k* to the actual distance unit, calculated as $d_{i}=c/f_{s}\times k$, $d_{i}$, gives the distance from the *i*-th radar to the target measured.

The position of the target can be obtained by using the obtained $d_{i}$ and the least-squares (LS) method. To apply LS, it is necessary to change the equation to be more simple. The circle equation, with radius $d_{i}$ centered on the location of the *i*-th radar, $\left(x_{i},y_{i},z_{i}\right)$ is represented by

(7) $${\left(x-x_{i}\right)}^{2}+{\left(y-y_{i}\right)}^{2}+{\left(z-z_{i}\right)}^{2}=d_{i}^{2}.$$

Equation (7) for the *l*-th radar and the *m*-th radar can be rearranged, as Equation (8), to convert the quadratic equation into a linear equation:

(8) $$2x\left(x_{m}-x_{l}\right)+2y\left(y_{m}-y_{l}\right)+2z\left(z_{m}-z_{l}\right)=d_{l}^{2}-d_{m}^{2}-x_{l}^{2}+x_{m}^{2}-y_{l}^{2}+y_{m}^{2}-z_{l}^{2}+z_{m}^{2}.$$

To obtain the solution in the LS scheme, we can convert Equation (8) to matrix equation form, Ax=b, where A and *b* can be expressed as

(9) $$A=2\left[\begin{array}{ccc} x_{1}-x_{0} & y_{1}-y_{0} & z_{1}-z_{0}\\ x_{2}-x_{1} & y_{1}-y_{0} & z_{1}-z_{0}\\ \cdot \cdot \cdot & \cdot \cdot \cdot & \cdot \cdot \cdot \\ x_{N_{r}}-x_{N_{r}-1} & y_{N_{r}}-y_{N_{r}-1} & z_{N_{r}}-z_{N_{r}-1} \end{array}\right],\;b=\left[\begin{array}{c} C_{1}\\ C_{2}\\ \cdot \cdot \cdot \\ C_{N_{r}} \end{array}\right].$$

$C_{i}$ replaces the right side of Equation (8) as $d_{i}^{2}-d_{i-1}^{2}-x_{i}^{2}+x_{i-1}^{2}-y_{i}^{2}+y_{i-1}^{2}-z_{i}^{2}+z_{i-1}^{2}$. As the solution of LS is well known as the right side of Equation (10), we can obtain the position $p={\left[x_{t},y_{t},z_{t}\right]}^{T}$ of the target:

(10) $$p={\left(A^{T}A\right)}^{-1}A^{T}b.$$

Position data can be obtained in real-time using the positioning method mentioned above. The position data for time *n* can be represented as $p\left[n\right]={\left[x_{t}\left[n\right],y_{t}\left[n\right],z_{t}\left[n\right]\right]}^{T}$, where $x_{t}\left[n\right]$, $y_{t}\left[n\right]$, and $z_{t}\left[n\right]$ are the three-dimensional coordinates of the target. Using numerical differentiation, the velocity and acceleration of the target can be expressed as:

(11) $$\begin{array}{cc} v[n] & =\left(p[n]-p[n-1]\right)/t_{r}\\ a[n] & =\left(v[n]-v[n-1]\right)/t_{r}. \end{array}$$

The observation period $t_{r}$ of the radar is the sampling period for the target position data. The initial value can be specified by v[0]=v[1]=a[0]=0. However, a[n] will be closer to the acceleration at n−1, and not exactly at *n*. If the $t_{r}$ is sufficiently small, there will be little time difference between *n* and n−1, and delay by one sample will not have a large impact. Therefore, even if the acceleration is not accurate, the acceleration and speed are calculated with Equation (11) to maintain real-time processing. As a result, the activity indicator for the spatial movement can be represented as:

(12) $$M_{spatial}\left[n\right]=\beta \cdot a\left[n\right]=\gamma \left(p\left[n\right]-2p\left[n-1\right]+p\left[n-2\right]\right).$$

As the amount of activity is not mathematically defined, the goal is not to create an accurate mathematical model in this paper. In other words, our goal is not to prove the exact relationship between acceleration and activity, but rather to suggest an indicator for objectively comparing activity. Therefore, we modeled the amount of activity and acceleration as a linearly proportional relationship.

## 4. Experiment Results

**Figure 2:**
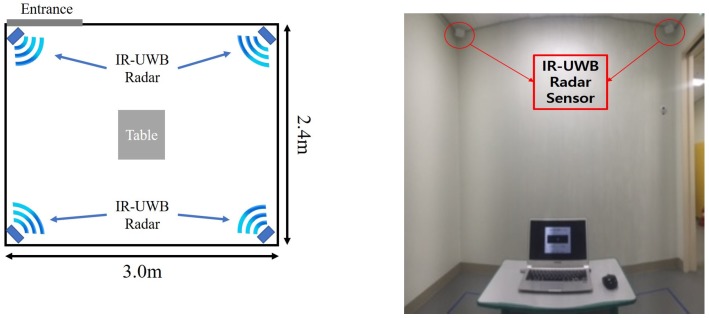
Experimental environment.

The XK300-MVI (Xandar Kardian, Toronto, ON, Canada) radar was used to verify the above algorithm. The X4M03 can select various center frequencies, from 7.29 GHz to 8.748 GHz, by adjusting various parameters according to local regulations. In these experiments, we selected the parameter with a center frequency of 8.748 GHz and a bandwidth of 1.5 GHz as −10 dB concept. The radiation power of the radar was 68.85 μW. The radar receiver can sample at 23.328 GS/s. The four radars were installed in each ceiling corner of the experimental room, as shown in Figure 2; which was 2.4 m in width, 3.0 m in length, and 2.4 m in height. In the indoor space, a distance error may occur due to the volume of a person; the radars were installed radially to minimize this error. The signal from the radars can be disturbed by movement of the arms or legs of the target. Hence, in order to reduce the effect of limbs as much as possible, radars were installed on the ceiling to observe the target. All of these radars were connected to the PC through the USB interface. The signal frames received from the radar were converted into digital values and transmitted to the PC by USB. These data were processed in MATLAB 2018b using the signal processing algorithm. The operating system of the PC was Windows 10. The frame-per-second (FPS) value of the signal received by the radar was 30.

A table and a laptop were placed in the middle of the room for the experiment. In Scenario 1, the tester focused on the notebook. Scenarios 2 and 3 were also measured for sedentary movement situations, sitting in front of the table. From the center table, a space of approximately 1.2 m by 1.2 m was designated as a narrow area for Scenarios 4 and 6, and the entire room area was designated as a wide area for Scenarios 5 and 7. All of the experimenters performed scenarios consecutively, and they acted in a condition for about three minutes per scenario. Additionally, the empty room was used to generate the threshold value by measuring data for about three minutes.

The actigraphy data was measured, for comparison with the proposed IR-UWB radar base. Actigraphy sensors wGT3X-BT (ActiGraph, Florida, US) were worn on the right wrist and right ankle. In each actigraphy sensor, there is an accelerometer for measuring the movement of the body part. With a dedicated-license software called ActiLife (Actigraph, Florida, US), vector magnitude values were extracted to the PC at a sampling rate of 1 s. The data of the actigraphy sensors were scaled to compare the trends.

### 4.1. Experiment Results for Each Scenario

**Figure 3:**
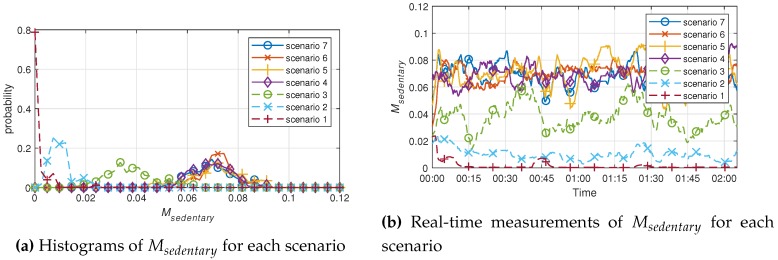
Experimental results of the sedentary movement index, $M_{sedentary}$, for each scenario.

During the experiment, it was possible to check the data in real time. However, the distribution of results is more useful to characterize each scenario. Five researchers participated in the experiment, and were assigned three minutes per scenario. The results of applying the algorithm for sedentary movement are shown in Figure 3. The experimental results of $M_{sedentary}$ for Scenarios 1–3 are shown, with a significant difference, in Figure 3a. The experimental results show that $M_{sedentary}$ values increased in the order of Scenarios 1–3. Individual behaviors may vary in the same scenario, so the outcome varied slightly for each person. However, the trends in the results were all similar. As the scenario progressed from 1 to 3, the value of $M_{sedentary}$ increased, which indicates that the results of each scenario were consistent and relevant. Conversely, in Scenarios 4 to 7, the results for spatial movement showed no significant difference in histogram and real-time measurement, and the values tended to be similar. This is effective for distinguishing the degree of sedentary movement with the algorithm used in Section 3.1, but it is insufficient for judging the degree of spatial movement around the room.

**Figure 4:**
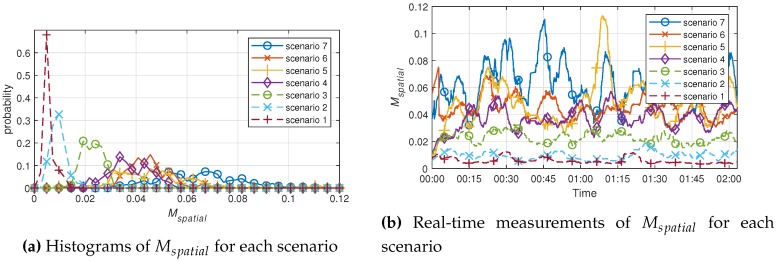
Experiment results of the spatial movement index, $M_{spatial}$, for each scenario.

In Section 3.2, the algorithm to find the position and acceleration of the subject was applied to the seven scenarios. As a result, the algorithm was made based on changes in the target’s position, so Scenarios 1 to 3 were smaller than Scenarios 4 to 7, and were not well distinguished. As the motions corresponding to Scenarios 4 to 7 consisted of traveling around the inside of the experimental room, the velocity wes not constant, and the acceleration varied instantaneously. Therefore, the variation of the $M_{spatial}$ value for Scenarios 4 to 7 was larger than that of Scenarios 1 to 3. Additionally, Scenarios 5 and 7 consisted of traveling around the lab within a large radius, and are generally larger than Scenarios 4 and 6, which consisted of traveling within a small radius, as can be identified in Figure 4. Scenario 7, which involved moving quickly within a large radius, had the highest value in most real-time measurement areas. Each scenario can be distinguished by the $M_{spatial}$ value derived from the algorithm used, and it was shown that the degree of movement can be presented by measuring the spatial movement using the suggested indicator.

The mean values of each scenario obtained from the sedentary movement indicator, $M_{sedentary}$, and spatial movement indicator, $M_{spatial}$, are shown in Table 1 and Table 2. It is difficult to compare the values of $M_{sedentary}$ and $M_{spatial}$ by themselves, because the algorithms used were different, and there are no units.

Looking at the results in Table 1 and Table 2, it can be seen that, for the same target scenario, the other targets were measured to be similar. Under similar circumstances, the measurement results are expected to be obtained at constant values. In the case of the $M_{spatial}$ of Scenarios 2 and 3, we can see that there is a significantly greater difference than for Scenarios 1 and 2. Scenarios 1–3 are all cases of sedentary movement, but position change was observed as much as torso shaking in Scenario 3. Nonetheless, the sedentary movement indicator is better discriminated in Scenarios 1–3. On the other hand, the spatial movement indicator is unlikely to have a sense of value in real-time, though it statistically has a significant difference in all scenarios. Sedentary movement indicators in Scenario 7 were measured to be lower than in Scenario 6, but more than or similar to those in Scenario 4. This clearly shows that a sedentary movement indicator cannot distinguish a change in position. However, from a different point of view, it can be seen that all walking scenarios show a certain value. In other words, we can see that Scenarios 4–7 can be classified as similar situations, because they do not observe a change in position from the point of view of the sedentary movement indicator. This confirms that similar behavior is measured at similar values.

The physical conditions of the researchers participating in the measurement are shown in Table 3. Although physical conditions did not differ greatly from each other, they do not seem to have a significant effect on the results. However, it is expected that $M_{sedentary}$ will be measured as small for small children. Because their size is small, their motion is also small. Conversely, it is expected that there will be no significant difference because $M_{spatial}$ depends on position changes.

### 4.2. Comparison with Actigraphy

**Figure 5:**
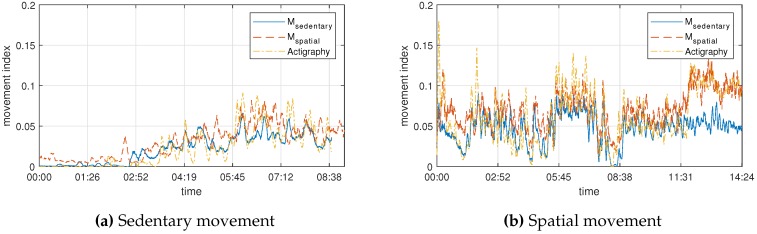
Graph of results when measured simultaneously with actigraphy. (**a**) and (**b**) are the results for sedentary and spatial movement, respectively. Actigraphy was scaled because the unit of the result data was not an actual physical quantity.

The proposed index was verified using an actigraphy sensor, which is actually used for clinical activity measurement. Similarly, we proceeded with seven scenarios, with a graph comparing the changes in real time to confirm the similarity of the data, as shown in Figure 5. Both sedentary and spatial movements can be seen to fit well with the actigraphy data. However, Figure 5b shows that, for the last 3 min, only the sedentary movement indicator is different. This is explained in Section 4.1—because the actigraphy sensor is similar to the acceleration for spatial movement index calculation, it can be seen that $M_{sedentary}$ and actigraphy are very similar.

**Figure 6:**
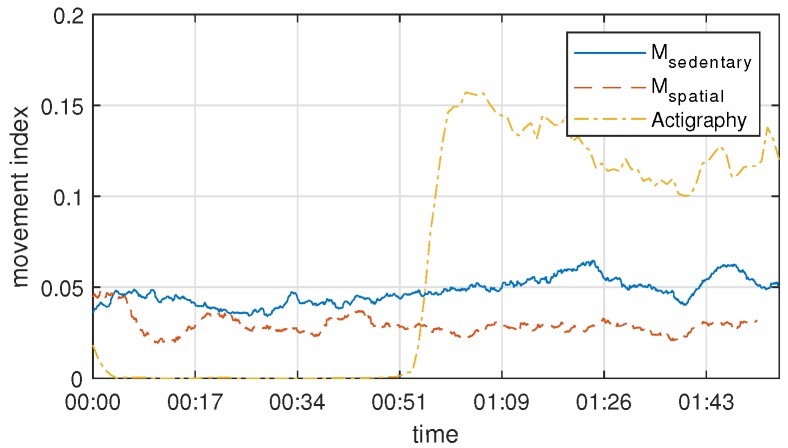
An extreme example of the proposed method and the use of actigraphy sensor. The actigraphy sensor is worn on a specific part of the body, so it may not reflect the whole movement (the first 50 s) or over-reflected (the last 50 s).

The limitations of the actigraphy sensor, compared with the radar sensor, can be seen in Figure 6, through an experiment of two extreme cases. In the previous 50 s, there was motion in the opposite hand and foot with the actigraphy sensor and, for the 50 s thereafter, there was movement of the hands and feet wearing the actigraphy sensor. The amount of movement measured from the radar during the entire section is largely constant, but the first 50 s has little movement measured from the actigraphy sensor. This result shows that the actigraphy sensor can only detect movement of a specific body part, but the radar sensor can detect movement of the entire body, even though it cannot distinguish each body part.

The actigraphy sensor is a contact-type sensor that should be worn on the wrists and ankles of the subject, and this can cause some pressure or stress for the subject during the experiment. In contrast, radar sensors are able to observe the patient’s movements in a non-contact manner and so do not disturb the subject. It can be said that a radar sensor, which is a non-contact sensor, is effective for obtaining more detailed and reliable data in the diagnosis of movement disorders. In addition, the actigraphy sensor informs the activity amount of the subject, but it does not provide the direction of that amount of activity, the location of the actual subject, or the movement route. The position of the subject, obtained from multiple radar sensors, can provide more information than the actigraphy sensor in diagnosing a subject’s specific habits, abrupt behavior, or hyperactivity. To date, there has been no index that can objectively express hyperactivity or specific movements in patients with movement disorders. However, it is possible to determine the position of the subject using the proposed algorithm and the radar sensor, and to measure the subject’s degree of movement from the objectively quantified indicator.

## 5. Conclusions

This paper proposed an algorithm and indicators to measure the amount of activity of people observed within certain constraints. Specifically, human activity was categorized into two types—with and without location movement—and two measurement indicators were proposed, that are specific to each activity with the signal strength and distance value data, measured by the radar sensor. Several scenarios and commercial products were used to confirm the reliability of the proposed indicators. Measurement can be performed simultaneously with the existing inspection to identify the actual activity amount, and this will be helpful in comparing the activity amount because the activity is quantified and more objective data is obtained. The IR-UWB radar sensors can measure heart rate and breathing while also recognizing gestures, making it a more practical solution in the medical field as it can be extended for additional functions in the future. Therefore, this quantitative technology for activity measurement may be useful in clinical applications as an assistive (complimentary tool) to diagnose movement disorders and to evaluate the efficacy of treatment based on direct observation by psychiatrists. It can additionally be used to overcome the limitations of conventional questionnaires by providing objective kinematic information about patients with movement disorders. Further, we can select indicators that are appropriate for our purpose because we can derive additional indicators (distance measurement, classification of standing and standing conditions, measuring activity area, among others) from our proposed algorithm.

## Figures and Tables

**Table 1:** Numerical results are shown for each target. The results for the $M_{sedentary}$ indicator are summarized (no unit).

Scenario	Mean of $M_{\mathrm{sedentary}}$					
	A	B	C	D	E	Total
1	0.16	0.16	0.14	0.30	0.19	0.19
2	1.01	1.77	0.76	0.73	0.85	1.02
3	3.81	4.27	2.30	2.20	2.01	2.92
4	6.99	5.10	2.78	5.72	4.74	5.07
5	7.11	5.04	4.64	5.11	4.43	5.27
6	7.15	6.60	3.06	5.04	7.02	5.77
7	6.94	5.39	4.57	3.85	6.25	5.40

**Table 2:** Numerical results are shown for each target. The results for the $M_{spatial}$ indicator are summarized (no unit).

Scenario	Mean of $M_{\mathrm{spatial}}$					
	A	B	C	D	E	Total
1	0.55	0.68	0.56	0.54	0.47	0.56
2	0.95	1.25	1.20	1.72	1.56	1.34
3	2.28	2.65	2.89	2.53	3.46	2.76
4	3.71	2.52	2.75	3.75	2.91	3.13
5	4.77	4.27	4.56	5.37	3.40	4.47
6	4.54	3.21	4.15	5.82	3.44	4.23
7	6.46	6.16	5.45	7.55	5.60	6.24

**Table 3:** Physical condition of the participants.

Participants	A	B	C	D	E
Gender (M/F)	M	M	M	F	M
Height (cm)	174	176	167	171	177
Weight (kg)	75	67	65	63	90
